# Supplementary material for: Deciphering the genetics and mechanisms of predisposition to multiple myeloma
Source: Nat Commun. 2024 Aug 5;15:6644. doi: 10.1038/s41467-024-50932-7 (PMC11300596; doi:10.1038/s41467-024-50932-7)
Supplement: Supplementary file 1 — Supplementary Information [file 41467_2024_50932_MOESM1_ESM.pdf]

Supplementary Figure 1

Hallmarks of MM: abnormal plasma cells in the bone marrow (left); and lytic bone lesions (right). Additionally, patients with MM usually exhibit a monoclonal immunoglobulin ("M-protein") in the blood, made by the plasma cell clone. Common complications include anemia, thrombocytopenia, compromised immunity, pain, fractures, and hypercalcemia.

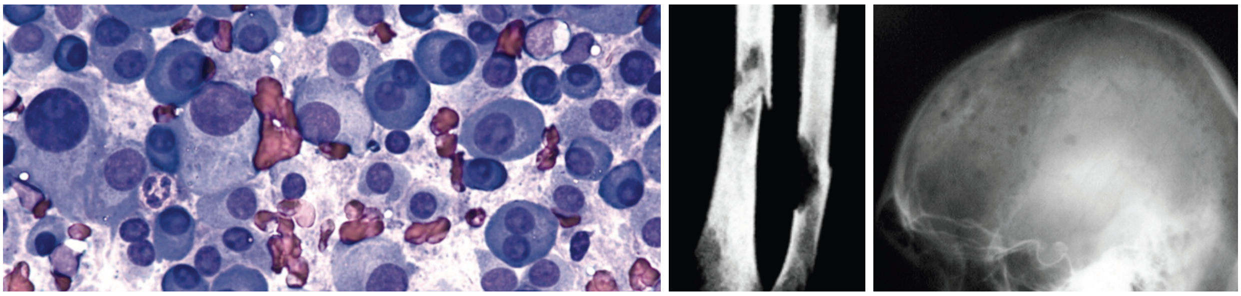

Supplementary Figure 2

Quantile-quantile plot of association statistics for the meta-analysis of the ten data sets, showing no evidence of substantial overinflation (λ=1.05, λ_{1000}=1).

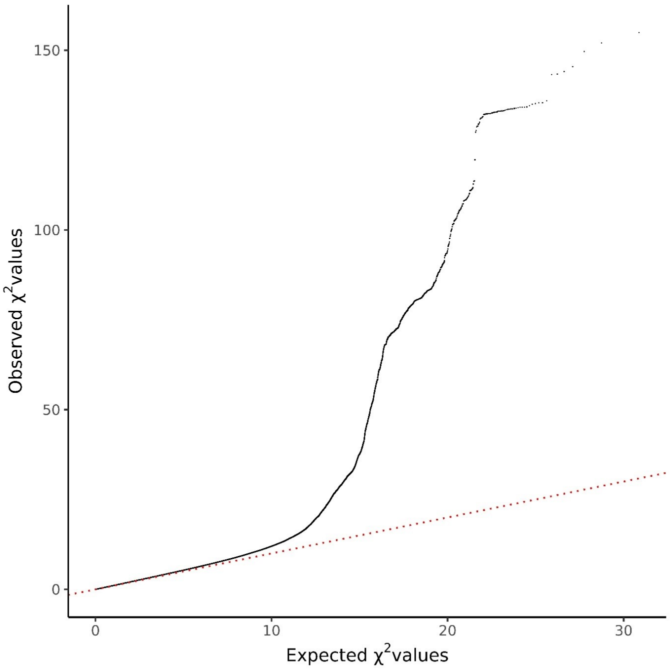

Supplementary Figure 3

Enrichment of histone marks in MM cell lines at MM risk variants. We observed an enrichment of risk variants in regions of active chromatin (H3K27ac, H3K4Me3 and H3K4Me1). The red line indicates the Bonferroni-adjusted P-value threshold.

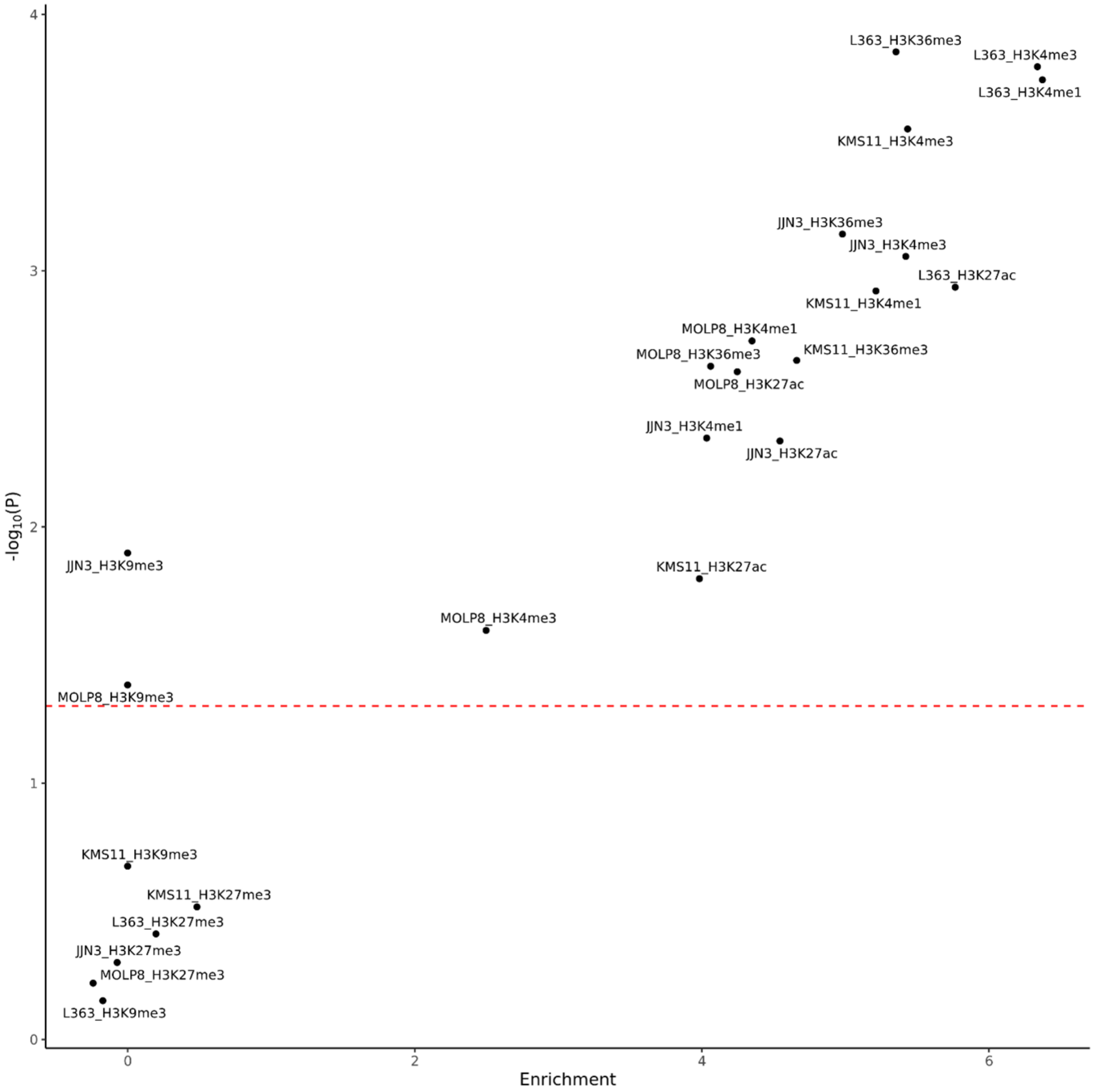

Supplementary Figure 4

Distributions of polygenic risk scores across different geographic populations. The distributions were estimated using the risk allele frequencies observed in our study (Study) and the five super-populations in the 1,000 Genomes compendium: European (EUR), Admixed American (AMR), African (AFR), East Asian (EAS), and South Asian (SAS). Within each population, we simulated 10,000 individuals under the assumption of independent, additive effects and equal effect sizes in all populations. For each simulated individual i, we calculated an overall raw score

$$\mathrm{PRS}_i = \prod_{j=1}^{35} e^{\beta_j * g_{ij}}, \quad (1)$$

where g_{ij} denotes the number of risk alleles at the j:th locus in the individual and β_j the study effect size for that locus. These scores were then normalized relative to the median raw score of our study population. Consistent with the higher incidence of MM in individuals of African and African-American ancestry, we observed the highest risk scores in the AFR super-population, which is explained by a higher prevalence of some risk alleles (**Supplementary Table 8**).

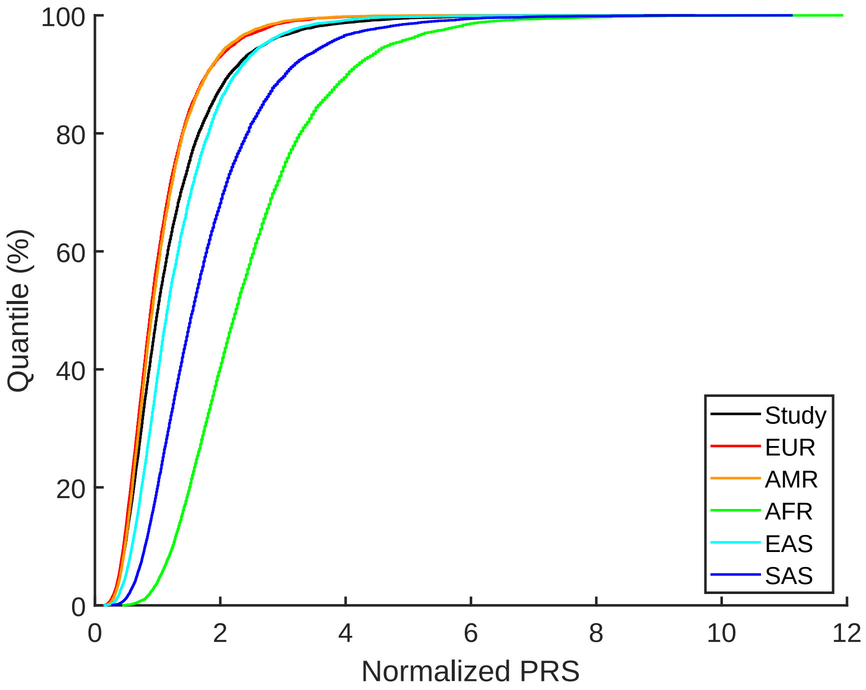

Supplementary Figure 5

Target gene expression in hematopoietic cell types. **(a)** Bulk mRNA-seq data. **(b)** Pseudobulked single-cell mRNA-seq data for 35,8552 blood and bone marrow cells. The color scale indicates log2 ratio relative to the median for each gene. **(c,d)** Enrichment of expression in individual cell types in the two data sets. Statistics are for Student's t-test for normalized expression values for one cell type versus the other cell types.

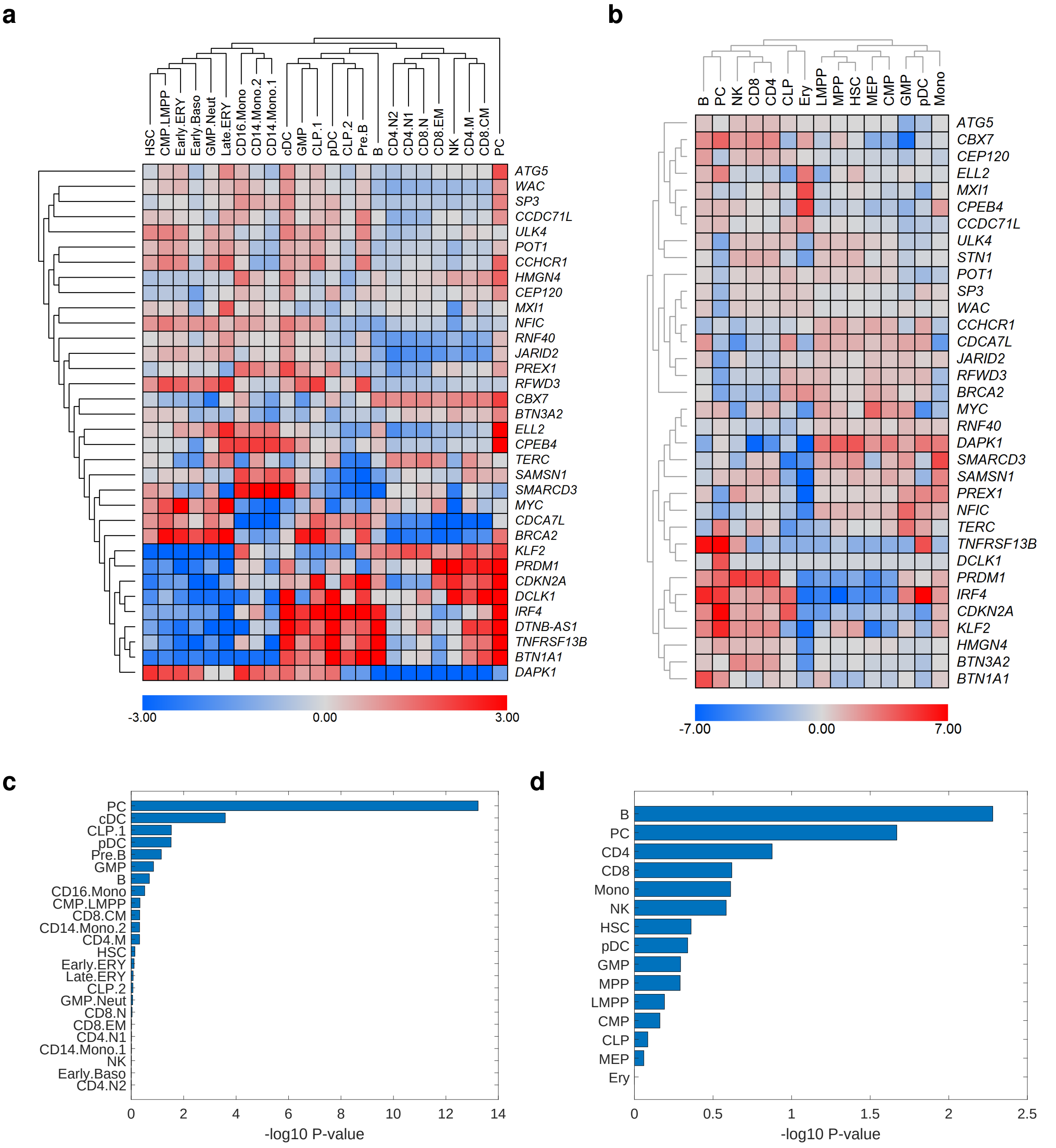

Supplementary Figure 6

Functional interactions between proteins encoded by target genes identified at MM loci using STRING (www.string-db.org). Line thickness indicates interaction confidence. The set of target genes showed more functional interactions than expected by chance (33 *vs.* 7 expected; P-value = 4.11×10^{-11}), with MYC as the main hub in the interaction network.

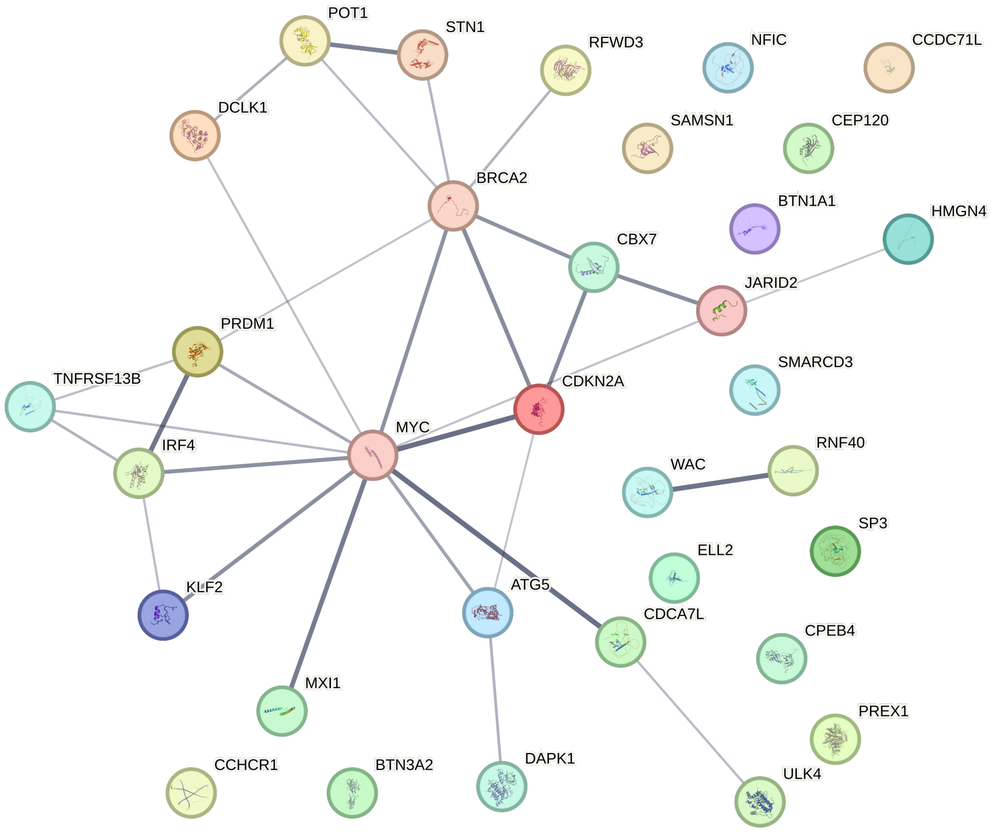

Supplementary Figure 7

Expression of *IL5RA* and *TNFRSF17* (BCMA) in hematopoietic cell types. **(a)** Bulk mRNA-seq data. **(b)** Pseudobulked single-cell mRNA-seq data for 35,8552 blood and bone marrow cells. The color scale indicates log2 ratio relative to the median for each gene.

a

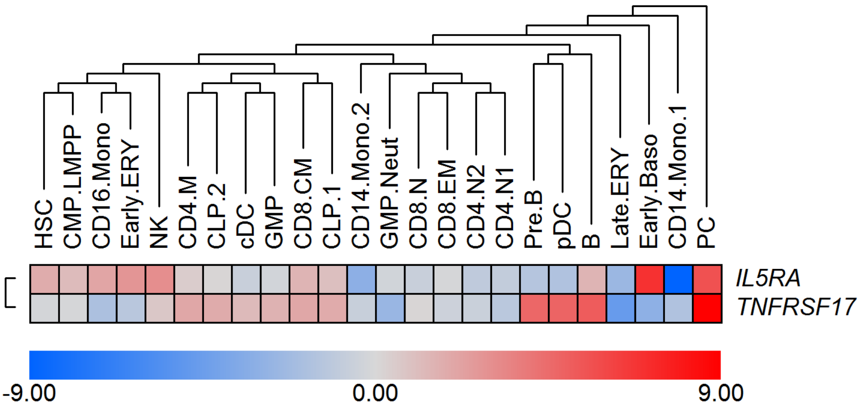

c

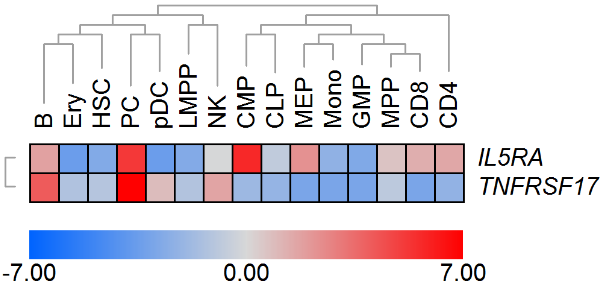

Supplementary Figure 8

Confirmation of dual-sgRNA CRISPR/Cas9 deletion efficiency. Raji cells were transfected with: (i) empty vector; (ii) non-targeting control; or (iii) pairs of sgRNAs generating deletions harboring either rs4273077 (approximately 108 bp) or r4792800 (approximately 143 bp). Lanes labeled "not used" represent sgRNA pairs that were not included in the final experiments.

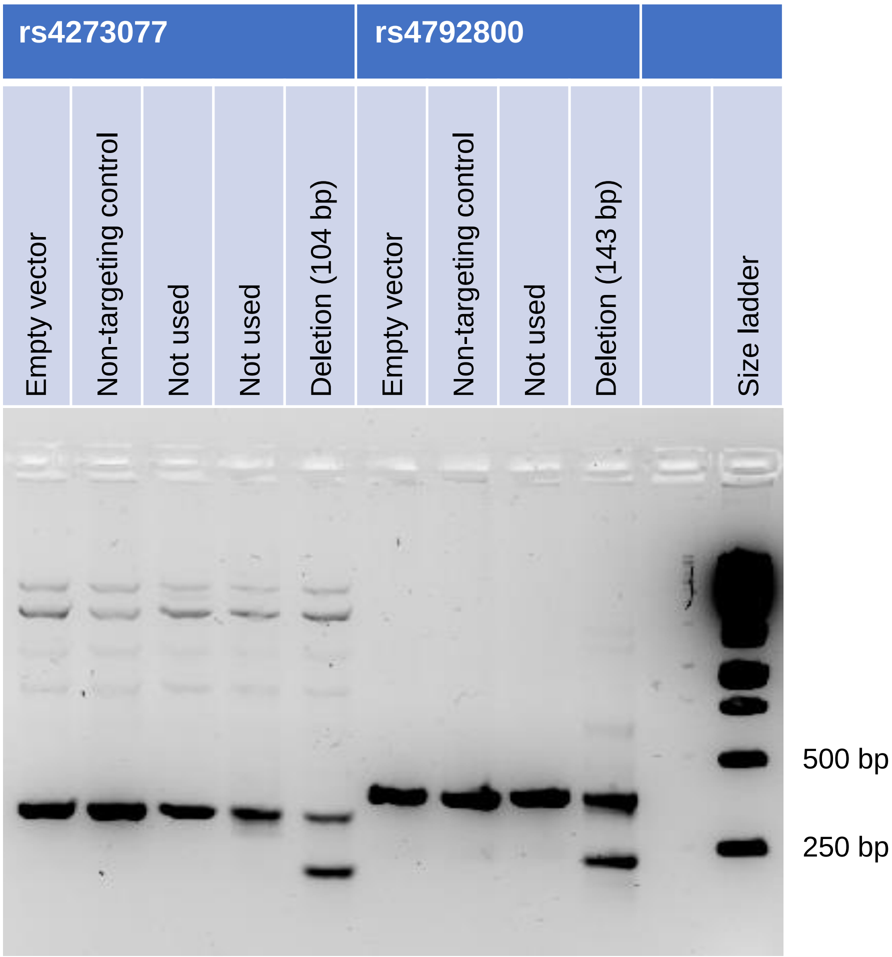

SUPPLEMENTARY NOTES

Deciphering the genetics and mechanisms of predisposition to multiple myeloma

Functional annotation and references for target genes implicated at each locus.

Table of contents

2p23.3 *DTNB-AS1* 3
2q31.1 *SP3* 3
3p22.1 *ULK4* 3
3q26.2 *TERC* 3
5q15 *ELL2* 4
5q23.2 *CEP120* 4
5q35.2 *CPEB4* 4
6p25.3 *IRF4* 5
6p22.2 *HMGN4*, *BTN1A1* and *BTN3A2* 5
6p22.3 *JARID2* 6
6p21.33 *HLA* region and *CCHCR1* 6
6q21 *ATG5* and *PRDM1* 6
7p15.3 *CDCA7L* 6
7q22.3 *CCDC71L* 7
7q31.33 *POT1* 7
7q36.1 *SMARCD3* 7
8q24.21 *MYC* 8
9p21.3 *CDKN2A* 8
9q21.3 *DAPK1* 8
10p12.1 *WAC* 9
10q25.2 *MXI1* 9
10q24.3 *STN1* 9
13q13.1 *BRCA2* 10
13q13.31 *DCLK1* 10
16p11.2 *RNF40* 10
16q23.1 *RFWD3* 10
17p11.2 *TNFRSF13B* 11
19p13.11 *KLF2* 11
20q13.1 *NFIC* 11
20q13.13 *PREX1* 12
21q11.2 *SAMSN1* 12
22q13.1 *CBX7* 12

2p23.3 *DTNB-AS1*

At 2p23.3, we identified the *DTNB-AS1* long non-coding RNA as a target gene based on an eQTL in plasmablasts and switched memory B-cells, with the risk allele conferring increased expression (**Supplementary Table 8**). As an anti-sense RNA, *DTNB-AS1* may be involved in regulation of DTNB (β-dystrobrevin), a component of the dystrophin-associated protein complex, which is important for cell structure and cell signalling.

2q31.1 *SP3*

The 2q31.1 MM risk allele upregulates *SP3* (Sp3 transcription factor) in plasma cells (**Supplementary Table 8**). SP3 has an established role in B-cell development influencing the expression of germinal centre genes, including activation-induced cytidine deaminase[1].

3p22.1 *ULK4*

The 3p22.1 association spans ULK4 (Unc-51 Like Kinase 4). ULK4 encodes a serine/threonine-protein kinase. Although the exact function of ULK4 is not known, the Atg1-ULK complex with ULK1 and ULK2 regulates mTOR-mediated autophagy, a pathway critical in MM biology[2,3]. The risk allele upregulates ULK4 in multiple hematopoietic cell types, including plasmablasts and switched memory B-cells (**Supplementary Table 8**). The MWAS data revealed decreasing methylation near ULK4 was associated with increasing MM risk. Correlated variants at this locus sit in the TSS and have looping to the gene, with corresponding ChromHMM annotations for promoter and enhancers respectively.

3q26.2 *TERC*

At 3q26.2, the variant rs2293607 (r2=0.81 with the lead variant rs7621631) map to the expressed sequence of *TERC* (telomerase RNA component). This long non-coding RNA is a

key component of telomerase, where it serves as a template for telomere replication. Colocalization of the signals at this locus between LTL and MM adds additional evidence to the idea that TERC is the candidate gene at this locus.

5q15 *ELL2*

ELL2 (elongation factor, RNA polymerase II 2) encodes a key component of the super-elongation complex, which mediates rapid gene induction by suppressing RNA polymerase II pausing[4]. In mature and memory B-cells, which express *ELL2* at a low level, Ig heavy chain (*IGH*) mRNA is translated to membrane-bound Ig[5]. In plasma cells, however, *ELL2* is highly expressed and helps RNA polymerase II find a promoter-proximal weak poly(A)-site, allowing *IGH* mRNA to be translated to secreted Ig[4,6-8]. The MM risk allele downregulates *ELL2* in plasma cells (**Supplementary Table 8**)[9,10]. MPRA revealed six putative causal variants (rs1458018, rs17085266, rs3777182, rs3777183, rs3777189, rs889302; **Table 2**). Only three of these have been previously reported, indicating that the mechanisms of the *ELL2* MM risk allele are more complex than previously believed[9-11].

5q23.2 *CEP120*

Variants at this locus reside in enhancer regions and at the TSS of *CEP120* (centrosomal protein of 120 kDa). In the TWAS, increased *CEP120* expression associated with increased MM risk (**Supplementary Table 3**). *CEP120* is required for microtubule assembly. Overexpression of *CEP120* has been reported to lead to uncontrolled centriole elongation[12].

5q35.2 *CPEB4*

At 5q35.2, we identified *CPEB4* (cytoplasmic polyadenylation element binding protein 4) as a target gene based on an eQTL in granulocytes (**Supplementary Table 8**), with the MM risk

allele yielding lower expression. Reporter assays identified rs72810983 and rs144869372 as putative causal variants (**Table 2**). rs72810983 sits at the *CPEB4* transcription start site; rs144869372 maps to an enhancer 4.9 kb upstream of CPEB4 with looping to the transcription start site. CPEB4 influences mRNA metabolism by binding to uridine-rich elements (consensus sequence 5'-UUUUUAU-3') within 3'-UTRs[13].

6p25.3 *IRF4*

The 6p25.3 associations maps to *IRF4* (interferon regulatory factor 4), which encodes a transcription factor crucial for the development and survival of MM plasma cells[14]. Recurrent somatic genetic lesions in *IRF4* have been reported in MM.

6p22.2 *HMGN4, BTN1A1* and *BTN3A2*

At 6p22.2, we identified *HMGN4* (High mobility group nucleosome-binding domain-containing protein 4), which encodes a nuclear protein that enhances gene transcription by reducing chromatin compactness[15]. The MM risk allele upregulates *HMGN4* in B-cells (**Supplementary Table 8**). Congruent with this, we identified the lead variant rs34565965 as a putative causal variant located in an enhancer element with looping to *HMGN4* (**Table 2** and **Supplementary Table 7**). At 6p22.2, we also identified BTN1A1 and BTN3A2 as target genes. These genes encode proteins of the immunoglobulin family previously implicated in the regulation of T-cell adaptive immunity and anti-tumour responses[16]. The MM risk allele downregulates *BTN1A1* in B-cells (**Supplementary Table 8**) and *BTN3A2* shows increasing expression with increasing MM risk in the TWAS (**Supplementary Table 3**).

6p22.3 *JARID2*

The 6p22.3 association is located in the promoter of *JARID2* (jumonji, AT-rich interactive domain 2). We identified this gene based on proximity, as we could not find any evidence of altered expression of *JARID2* in the available datasets, possibly because of the low allele frequency of this MM risk allele. JARID2 functions as a transcriptional repressor through recruitment of Polycomb repressive complex 2. It has been identified as a regulator of haematopoietic stem cells[17], and the 6p22.3 region is commonly gained in MM tumours[18].

6p21.33 *HLA* region and *CCHCR1*

The HLA region contains numerous genes relevant to B-cell function. While functional fine-mapping of this region is difficult due to extreme allelic variation, we identified *CCHCR1* (Coiled-coil alpha-helical rod protein 1) as a target gene based on an eQTL in B-cells, with the MM risk allele downregulating *CCHCR1* (**Supplementary Table 8**). *CCHCR1* modulate mRNA metabolism through interactions with mRNA-decapping proteins[19].

6q21 *ATG5* and *PRDM1*

At 6q21, we identified *ATG5* and *PRDM1* as target genes based on eQTLs in plasmablasts (**Supplementary Table 8**), with the MM risk allele downregulating both genes. *ATG5* is essential for plasma cell autophagy and survival[20-22]. *PRDM1* encodes a transcriptional repressor[23] with an established role in plasma cell development[24].

7p15.3 *CDCA7L*

CDCA7L (cell division cycle-associated 7-like protein) encodes a cell division-associated protein that binds the transcriptional co-activator p75 and potentiates MYC-mediated transformation events[25,26]. The MM risk allele upregulates *CDCA7L* in multiple blood cell

types, including plasma cells and switched B-cells (**Supplementary Table 8**)[27]. Consistent with this, the rs4487645 variant, which maps to an enhancer 3.6 kb downstream of CDCA7L, increases transcriptional activity in reporter assays by creating a novel IRF4 binding site [26]. We have previously validate the causality of rs4487645 by CRISPR/Cas9 editing[11].

7q22.3 *CCDC71L*

At 7q22.3, we identified CCDC71L (Coiled-Coil Domain Containing 71 Like) as a target gene based on TWAS data, where increased expression of *CCDC71L* associated with increased MM risk (**Supplementary Table 3**). The gene has been shown to promote cell proliferation, migration and invasion in breast cancer[28].

7q31.33 *POT1*

The 7q31.33 association maps upstream of *POT1* (protection of telomeres 1), encoding a key component of the shelterin complex that protects telomeres and chromosomal stability [29,30]. Colocalisation analysis between LTL and MM demonstrated a likely shared variant between traits at this locus. Moreover, rare loss-of-function variants in *POT1* have been reported in familial MM and familial chronic lymphocytic leukemia [31,32].

7q36.1 *SMARCD3*

At 7q36.1, we identified *SMARCD3* (SWI/SNF-related, matrix-associated, actin-dependent regulator of chromatin, subfamily d, member 3) as a target gene based on an eQTL in plasma cells (**Supplementary Table 8**). In humans, *SMARCD1*, *SMARCD2*, and *SMARCD3* encode alternative, mutually exclusive 60-kD subunits of the SWI/SNF nucleosome remodelling complex[33-36]. Among blood cells, *SMARCD3* is expressed in granulocytes and monocytes whereas its basal expression in plasma cells is very low; instead these cells show high

expression of *SMARCD1* and *SMARCD2*. By contrast, the MM risk allele upregulates *SMARCD3* ectopically in plasma cells. Using MPRA, we identified rs78740585 an rs73169649 as putative causal variants. Out of these, rs78740585 maps to an enhancer region with looping to the *SMARCD3* transcriptional start site, and creates an IRF4 binding motif[11].

8q24.21 *MYC*

At 8q24.21, we identified MYC as target gene based on a long-distance (0.5 Mb) chromatin looping interaction (**Supplementary Table 7** and **Table 2**) and an eQTL in naïve B-cells and plasmablasts, where the MM risk allele upregulates *MYC* (**Supplementary Table 8**). The MYC oncogene is commonly amplified or overexpressed in many cancers, including MM[37]. 8q24.21 has been associated with several other tumour types, including diffuse large-scale B-cell lymphoma[38], Hodgkin's lymphoma[39], and chronic lymphocytic leukaemia[40,41]. Using STRING, we found that the set of target genes showed more functional interactions than expected by chance (33 observed vs 7 expected; P=4.11x10^{-11}), with MYC as the main hub in the interaction network (**Supplementary Fig. 4**), indicating that the MYC-IRF4 pathway plays an important role in mediating inherited MM risk.

9p21.3 *CDKN2A*

The 9p21.3 association spans *CDKN2A* (cyclin-dependent kinase inhibitor 2A), a well-known tumor suppressor gene. Both germline and somatic variants at *CDKN2A* have been associated with several tumour types.

9q21.3 *DAPK1*

At 9q21.3, increasing expression of *DAPK1* (death associated kinase 1) associates with increasing myeloma risk in the patient TWAS (**Supplementary Table 3**). We also found

DAPK1 eQTLs in plasma cells and plasmablasts (**Supplementary Table 8**). Using reporter assays, we identified rs1329600 as a putative causal variant that sits in the transcriptional start site (**Table 2** and **Supplementary Table 7**). *DAPK1* is a regulator of cell death and autophagy, and acts as a component in the ER stress-induced cell death pathway[42].

10p12.1 *WAC*

The 10p12.1 associations maps to the autophagy gene *WAC* (WW domain-containing adapter protein with coiled-coil)[43]. Rare loss-of-function variants in *WAC* cause De Santo-Shinawi syndrome[44], which can feature low immunoglobulin levels[45]. The MM risk allele decreases WAC expression in plasma cells and plasmablasts (**Supplementary Table 8**). Using MPRA, we identified 2790444 as a putative causal variant (**Table 2**). This variant maps close to an intragenic enhancer region close to the transcription start site, and the MM risk allele rs2790444-T creates a binding site for the POU2F1 transcription factor[11].

10q25.2 *MXI1*

MXI1 (MAX-interacting protein 1) shows one of the strongest associations in the patient TWAS with decreasing expression increasing MM risk (**Supplementary Table 3**). Concordant with this, we also found an eQTL for *MXI1* in plasmablasts (**Supplementary Table 8**), with the MM risk allele yielding lower *MXI1* expression. Consistent with tumor suppressor activity, *MXI1* acts as a MYC antagonist by competing for MAX binding[46,47]. Somatic gene fusions involving *MXI1* have been reported in MM[48].

10q24.3 *STN1*

At 10q24.3, we identified *STN1* as a target gene based on eQTL in B-cells (**Supplementary Table 8**), with the MM risk allele yielding lower expression. *STN1* a component of the

shelterin complex subunit, which protects telomeres from DNA damage. Colocalization of the 10q24.3 signal between LTL and MM also supports *STN1* as a target gene at 10q24.3.

13q13.1 *BRCA2*

BRCA2 is a well-known tumor suppressor gene associated with several tumor types. The 13q13.1 association corresponds to a pathogenic truncating variant (Lys3326Ter)[49].

13q13.31 *DCLK1*

At 13q13.31,we identified *DCLK1* (doublecortin like kinase 1) as a target genes by TWAS, where increased *DCLK1* expression conferred increased MM risk (**Supplementary Table 3**). *DCLK1* encodes a serine/threonine kinase that has been extensively studied, and been shown to have oncogenic activity (reviewed in refs.[50,51]).

16p11.2 *RNF40*

The 16p11.2 spans several genes and we identified *RNF40* (E3 ubiquitin-protein ligase BRE1B) as a target gene based on eQTLs in hematopoietic cells (**Supplementary Table 8**). The encoded protein has been reported to be involved in histone modulation and to interact with, and affect the stability of, the DDB1-CUL4-based ubiquitin E3 ligase complex[52].

16q23.1 *RFWD3*

At 16q23.1, *RFWD3* (ring finger and WD repeat domain 3) exhibits eQTLs in plasma cells and plasmablasts (**Supplementary Table 8**). *RFWD3* encodes an E3 ubiquitin ligase that positively regulates p53 stability by forming an RFWD3–MDM2–p53 complex, thereby protecting p53 from degradation by MDM2-mediated polyubiquitination[53-55]. Biallelic loss-of-function mutations in *RFWD3* cause a Fanconi anemia-like disease phenotype[56].

17p11.2 *TNFRSF13B*

The 17p11.2 association spans TNFRSF13B (TNF receptor superfamily member 13B). This gene encodes the TACI receptor, which is a central regulator of B-cell responses and immunoglobulin class-switching. Loss-of-function mutations in TNFRSF13B associate with common variable immunodeficiency (CVID), a condition defined by low IgA and/or IgG levels due to stalled development of mature B-cells and plasma cells. An eQTL for increasing expression of *TNFRSF13B* in B-cells corresponded to the MM risk allele (**Supplementary Table 8**). Through MPRA, we identified rs4273077 and rs4792800 as putative causal gene-regulatory variants (**Supplementary Table 7**, **Table 2**, and **Fig. 4**). Additionally, the lead variant rs34562254 is a missense variant (Pro251Leu), which is predicted to be benign.

19p13.11 *KLF2*

The 19p13.11 locus harbors *KLF2* (Krüppel-like Factor 2), encoding a well-characterized transcription factor. Demethylation by KDM3A histone demethylase sustains KLF2 expression and influences IRF4-dependent MM cell survival[57]. The MM risk allele upregulates KLF2 in multiple hematologic cell types, including plasmablasts and B-cell subsets (**Supplementary Table 8**).

20q13.1 *NFIC*

At 20q13.1, we identified *NFIC* (Nuclear Factor 1-C) as a target gene based on an eQTL in whole blood, with the MM risk allele downregulating NFIC expression (**Supplementary Table 8**). The *NFIC* transcription factor is thought have tumor suppressor activity[58].

20q13.13 *PREX1*

PREX1 (phosphatidylinositol-3, 4, 5-trisphosphate-dependent Rac exchange factor 1) is a Rac guanine exchange factor that coordinates signalling inputs from G protein-coupled receptors and receptor tyrosine kinases. The MM risk allele upregulates PREX1 in plasma cells and memory B cells (**Supplementary Table 8**). PREX1 has previously been implicated in oncogenic signaling in breast cancer[59].

21q11.2 *SAMSN1*

At 21q11.2, we identified *SAMSN1* as a target gene based on an eQTL in (**Supplementary Table 8**), with the MM risk allele increasing its expression. *SAMSN1* encodes a regulator of B-cell activation. Interestingly, *SAMSN1* deletions have been reported in MM-prone mice[60]. Using reporter assays, we identified the lead variant rs2822736 as a putative causal variants that maps to an enhancer element in *SAMSN1* (**Table 2** and **Supplementary Table 7**).

22q13.1 *CBX7*

CBX7 (chromobox homolog 7) encodes a component of the polycomb repressive complex 1, which regulates cell fate determination and differentiation[61]. Interestingly, *CBX7* has been reported to mediate transcription repression on the *CDKN2A* tumor suppressor gene[62]. *CBX7* also cooperates with MYC to promote B-cell lymphomagenesis[63]. The MM risk allele upregulates *CBX7* in switched memory B-cells (**Supplementary Table 8**).

REFERENCES

1. Park, S.R. *et al.* HoxC4 binds to the promoter of the cytidine deaminase AID gene to induce AID expression, class-switch DNA recombination and somatic hypermutation. *Nat Immunol* **10**, 540-50 (2009).
2. Guglielmelli, T. *et al.* mTOR pathway activation in multiple myeloma cell lines and primary tumour cells: pomalidomide enhances cytoplasmic-nuclear shuttling of mTOR protein. *Oncoscience* **2**, 382-394 (2015).
3. Jung, C.H., Ro, S.-H., Cao, J., Otto, N.M. & Kim, D.-H. mTOR regulation of autophagy. *FEBS letters* **584**, 1287-1295 (2010).
4. Martincic, K., Alkan, S.A., Cheatle, A., Borghesi, L. & Milcarek, C. Transcription elongation factor ELL2 directs immunoglobulin secretion in plasma cells by stimulating altered RNA processing. *Nat Immunol* **10**, 1102-9 (2009).
5. Milcarek, C., Albring, M., Langer, C. & Park, K.S. The eleven-nineteen lysine-rich leukemia gene (ELL2) influences the histone H3 protein modifications accompanying the shift to secretory immunoglobulin heavy chain mRNA production. *J Biol Chem* **286**, 33795-803 (2011).
6. Shilatifard, A. *et al.* ELL2, a new member of an ELL family of RNA polymerase II elongation factors. *Proc. Natl. Acad. Sci. USA* **94**, 3639-3643 (1997).
7. Benson, M.J. *et al.* Heterogeneous nuclear ribonucleoprotein L-like (hnRNPLL) and elongation factor, RNA polymerase II, 2 (ELL2) are regulators ofmRNA processing in plasma cells. *proc. Natl. Acad. Sci. USA* **109**, 16252–16257 (2012).
8. Park, K.S. *et al.* Transcription Elongation Factor ELL2 Drives Ig Secretory-Specific mRNA Production and the Unfolded Protein Response. *J Immunol* (2014).

9. Li, N. *et al.* Genetic Predisposition to Multiple Myeloma at 5q15 Is Mediated by an ELL2 Enhancer Polymorphism. *Cell Rep* **20**, 2556-2564 (2017).

10. Ali, M. *et al.* The multiple myeloma risk allele at 5q15 lowers ELL2 expression and increases ribosomal gene expression. *Nat Commun* **9**, 1649 (2018).

11. Ajore, R. *et al.* Functional dissection of inherited non-coding variation influencing multiple myeloma risk. *Nat Commun* **13**, 151 (2022).

12. Yadav, S.P. *et al.* Centrosomal protein CP110 controls maturation of the mother centriole during cilia biogenesis. *Development* **143**, 1491-501 (2016).

13. Afroz, T. *et al.* A fly trap mechanism provides sequence-specific RNA recognition by CPEB proteins. *Genes Dev* **28**, 1498-514 (2014).

14. Shaffer, A.L. *et al.* IRF4 addiction in multiple myeloma. *Nature* **454**, 226-31 (2008).

15. Birger, Y., Ito, Y., West, K.L., Landsman, D. & Bustin, M. HMGN4, a newly discovered nucleosome-binding protein encoded by an intronless gene. *DNA Cell Biol* **20**, 257-64 (2001).

16. Afrache, H., Gouret, P., Ainouche, S., Pontarotti, P. & Olive, D. The butyrophilin (BTN) gene family: from milk fat to the regulation of the immune response. *Immunogenetics* **64**, 781-94 (2012).

17. Kinkel, S.A. *et al.* Jarid2 regulates hematopoietic stem cell function by acting with polycomb repressive complex 2. *Blood* **125**, 1890-900 (2015).

18. Walker, B.A. *et al.* A compendium of myeloma-associated chromosomal copy number abnormalities and their prognostic value. *Blood* **116**, e56-65 (2010).

19. Ling, Y.H. *et al.* CCHCR1 interacts with EDC4, suggesting its localization in P-bodies. *Exp Cell Res* **327**, 12-23 (2014).

20. Pengo, N. *et al.* Plasma cells require autophagy for sustainable immunoglobulin production. *Nat Immunol* **14**, 298-305 (2013).

21. Conway, K.L. *et al.* ATG5 regulates plasma cell differentiation. *Autophagy* **9**, 528-537 (2013).

22. Cenci, S. Autophagy, a new determinant of plasma cell differentiation and antibody responses. *Mol Immunol* **62**, 289-95 (2014).

23. Mitchell, J.S. *et al.* Genome-wide association study identifies multiple susceptibility loci for multiple myeloma. *Nat Commun* **7**, 12050 (2016).

24. Shapiro-Shelef, M. & Calame, K. Regulation of plasma-cell development. *Nat Rev Immunol* **5**, 230-42 (2005).

25. Tian, Y. *et al.* CDCA7L promotes hepatocellular carcinoma progression by regulating the cell cycle. *Int J Oncol* **43**, 2082-90 (2013).

26. Li, N. *et al.* Multiple myeloma risk variant at 7p15.3 creates an IRF4-binding site and interferes with CDCA7L expression. *Nat Commun* **7**, 13656 (2016).

27. Weinhold, N. *et al.* The 7p15.3 (rs4487645) association for multiple myeloma shows strong allele-specific regulation of the MYC-interacting gene CDCA7L in malignant plasma cells. *Haematologica* **100**(2015).

28. Luo, X. & Wang, H. LINC00514 upregulates CCDC71L to promote cell proliferation, migration and invasion in triple-negative breast cancer by sponging miR-6504-5p and miR-3139. *Cancer Cell Int* **21**, 180 (2021).

29. Pinzaru, A.M. *et al.* Telomere Replication Stress Induced by POT1 Inactivation Accelerates Tumorigenesis. *Cell Rep* **15**, 2170-2184 (2016).

30. Rice, C. *et al.* Structural and functional analysis of the human POT1-TPP1 telomeric complex. *Nat Commun* **8**, 14928 (2017).

31. Speedy, H.E. *et al.* Germ line mutations in shelterin complex genes are associated with familial chronic lymphocytic leukemia. *Blood* **128**, 2319-2326 (2016).

32. Hakkarainen, M. *et al.* A germline exome analysis reveals harmful POT1 variants in multiple myeloma patients and families. *EJHaem* **3**, 1352-1357 (2022).

33. Flajollet, S., Lefebvre, B., Cudejko, C., Staels, B. & Lefebvre, P. The core component of the mammalian SWI/SNF complex SMARCD3/BAF60c is a coactivator for the nuclear retinoic acid receptor. *Mol Cell Endocrinol* **270**, 23-32 (2007).

34. Wang, W. *et al.* Diversity and specialization of mammalian SWI/SNF complexes. *Genes Dev* **10**, 2117-30 (1996).

35. Mashtalir, N. *et al.* Modular Organization and Assembly of SWI/SNF Family Chromatin Remodeling Complexes. *Cell* **175**, 1272-1288 e20 (2018).

36. Puri, P.L. & Mercola, M. BAF60 A, B, and Cs of muscle determination and renewal. *Genes Dev* **26**, 2673-83 (2012).

37. Manier, S. *et al.* Genomic complexity of multiple myeloma and its clinical implications. *Nat Rev Clin Oncol* **14**, 100-113 (2017).

38. Cerhan, J.R. *et al.* Genome-wide association study identifies multiple susceptibility loci for diffuse large B cell lymphoma. *Nat Genet* **46**, 1233-8 (2014).

39. Enciso-Mora, V. *et al.* A genome-wide association study of Hodgkin's lymphoma identifies new susceptibility loci at 2p16.1 (REL), 8q24.21 and 10p14 (GATA3). *Nat Genet* **42**, 1126-30 (2010).

40. Crowther-Swanepoel, D. *et al.* Common variants at 2q37.3, 8q24.21, 15q21.3 and 16q24.1 influence chronic lymphocytic leukemia risk. *Nat Genet* **42**, 132-6 (2010).

41. Sud, A., Kinnersley, B. & Houlston, R.S. Genome-wide association studies of cancer: current insights and future perspectives. *Nat Rev Cancer* **17**, 692-704 (2017).

42. Martoriati, A. *et al.* dapk1, encoding an activator of a p19ARF-p53-mediated apoptotic checkpoint, is a transcription target of p53. *Oncogene* **24**, 1461-6 (2005).

43. Joachim, J., Wirth, M., McKnight, N.C. & Tooze, S.A. Coiling up with SCOC and WAC: two new regulators of starvation-induced autophagy. *Autophagy* **8**, 1397-400 (2012).

44. DeSanto, C. *et al.* WAC loss-of-function mutations cause a recognisable syndrome characterised by dysmorphic features, developmental delay and hypotonia and recapitulate 10p11.23 microdeletion syndrome. *J Med Genet* **52**, 754-61 (2015).

45. Vanegas, S., Ramirez-Montano, D., Candelo, E., Shinawi, M. & Pachajoa, H. DeSanto-Shinawi Syndrome: First Case in South America. *Mol Syndromol* **9**, 154-158 (2018).

46. Zervos, A.S., Gyuris, J. & Brent, R. Mxi1, a protein that specifically interacts with Max to bind Myc-Max recognition sites. *Cell* **72**, 223-32 (1993).

47. Armstrong, M.B. *et al.* N-Myc differentially regulates expression of MXI1 isoforms in neuroblastoma. *Neoplasia* **15**, 1363-70 (2013).

48. Lin, M. *et al.* Identification of novel fusion transcripts in multiple myeloma. *J Clin Pathol* **71**, 708-712 (2018).

49. Wang, Y. *et al.* Rare variants of large effect in BRCA2 and CHEK2 affect risk of lung cancer. *Nat Genet* **46**, 736-41 (2014).

50. Ye, L. *et al.* DCLK1 and its oncogenic functions: A promising therapeutic target for cancers. *Life Sci* **336**, 122294 (2023).

51. Lu, Q. *et al.* Role of DCLK1 in oncogenic signaling (Review). *Int J Oncol* **61**(2022).

52. Yadav, P. *et al.* M6A RNA Methylation Regulates Histone Ubiquitination to Support Cancer Growth and Progression. *Cancer Res* **82**, 1872-1889 (2022).

53. Elia, A.E. *et al.* RFWD3-Dependent Ubiquitination of RPA Regulates Repair at Stalled Replication Forks. *Mol Cell* **60**, 280-93 (2015).

54. Fu, X. *et al.* RFWD3-Mdm2 ubiquitin ligase complex positively regulates p53 stability in response to DNA damage. *Proc Natl Acad Sci U S A* **107**, 4579-84 (2010).

55. Inano, S. *et al.* RFWD3-Mediated Ubiquitination Promotes Timely Removal of Both RPA and RAD51 from DNA Damage Sites to Facilitate Homologous Recombination. *Mol Cell* **66**, 622-634 e8 (2017).

56. Knies, K. *et al.* Biallelic mutations in the ubiquitin ligase RFWD3 cause Fanconi anemia. *J Clin Invest* **127**, 3013-3027 (2017).

57. Ohguchi, H. *et al.* The KDM3A-KLF2-IRF4 axis maintains myeloma cell survival. **7**, 10258 (2016).

58. Zhang, H. *et al.* Transcription factor NFIC functions as a tumor suppressor in lung squamous cell carcinoma progression by modulating lncRNA CASC2. *Cell Cycle* **21**, 63-73 (2022).

59. Ryan, M.B. *et al.* ERK/MAPK Signaling Drives Overexpression of the Rac-GEF, PREX1, 2 in BRAF- and NRAS-mutant Melanoma. *Mol Cancer Res.* (2016).

60. Amend, S.R. *et al.* Whole Genome Sequence of Multiple Myeloma-Prone C57BL/KaLwRij Mouse Strain Suggests the Origin of Disease Involves Multiple Cell Types. *PLoS One* **10**, e0127828 (2015).

61. Gil, J., Bernard, D. & Peters, G. Role of polycomb group proteins in stem cell self-renewal and cancer. *DNA Cell Biol* **24**, 117-25 (2005).

62. Aguilo, F., Zhou, M.M. & Walsh, M.J. Long noncoding RNA, polycomb, and the ghosts haunting INK4b-ARF-INK4a expression. *Cancer Res* **71**, 5365-9 (2011).

63. Scott, C.L. *et al.* Role of the chromobox protein CBX7 in lymphomagenesis. *Proc Natl Acad Sci U S A* **104**, 5389-94 (2007).
