# Supplementary material for: Deciphering the genetics and mechanisms of predisposition to multiple myeloma
Source: Nat Commun. 2024 Aug 5;15:6644. doi: 10.1038/s41467-024-50932-7 (PMC11300596; doi:10.1038/s41467-024-50932-7)
Supplement: Supplementary file 3 — Description of Additional Supplementary Files [file 41467_2024_50932_MOESM3_ESM.pdf]

Description of Additional Supplementary Files

File Name: Supplementary Data 1

Description: Data sets used in the MM meta-analysis. Abbreviations: MyIX, Myeloma IX; MyXI, Myeloma XI; B-PROOF, B-vitamins for the prevention of osteoporotic fractures; UKGPCS, UK Genetic Prostate Cancer Study; BCAC, Breast Cancer Association Consortium.

File Name: Supplementary Data 2

Description: Summary statistics for novel variants for each datasets in the meta-analysis. Odds ratios derived with respect to the effect allele. Shown are discovery association GWAS betas and GWAS *P*-values for individual studies, as well as standard error (SE) and info score. P_{HET} is *P*-value for heterogenety. I^2 is measure of heterogeneity between datasets in meta-analysis. *Q* is Cochran's *Q*-statistic.

File Name: Supplementary Data 3

Description: Transcriptome-wide association study in $CD138^+$ plasma cells isolated from the bone marrow of MM patients (n=1,449). Detailed are S-MultiXcan *P*-values for association between gene expression and MM risk, along with the *Z*-scores quantifying this relationship (positive Z indicates increased gene expression increases risk). *N* and N_{indep} indicate the total number of single-cohort results used for S-MultiXcan analysis and the number of independent components after singular value decomposition, respectively. The horizontal line indicates the Bonferroni threshold for transcriptome-wide significance ($P < 2\times10^{-6}$).

File Name: Supplementary Data 4

Description: Methylome-wide association study in $CD138^+$ plasma cells from the bone marrow of MM patients (*n*=379). Detailed are the S-MultiXcan *P*-values for association with methylation and the Z-scores quantifying this relationship (positive *Z* indicates increased methylation increases risk). *N* and N^{indep} indicate the total number of single-cohort results used for S-MultiXcan analysis and the number of independent components after singular value decomposition. The horizontal line indicates the Bonferroni threshold for methylome-wide significance ($P < 2\times10^{-6}$).

File Name: Supplementary Data 5

Description: Associations between risk variants and MM subtypes defined by somatic acquired genetic lesions. Abbreviations: Other allele (OA), Effect allele (EA), Effect allele frequency (EAF).

File Name: Supplementary Data 6

Description: Relationship between genotype of MM lead variants and monoclonal gammopathy of undtermined significance (MGUS). Meta-analysis of 6,234 MGUS cases and 720,297 controls from Iceland (4,092 cases and 298,673 controls), UK Biobank (1,150 cases and 427,714 controls) and Germany (992 cases and 2,910 controls). Abbreviations: EA, effect allele; OA, other allele; EAF, effect allele frequency.

File Name: Supplementary Data 7

Description: Pleiotropic associations with diseases and immunological/hematological traits in the GWAS catalog (r^2>0.8 between trait lead variant and MM lead variant).

File Name: Supplementary Data 8

Description: Risk allele frequencies in different populations. Abbreviations: Risk allele frequency (RAF), European (EUR), African (AFR), Admixed American (AMR), East Asian (EAS), South Asian (SAS). Effect allele (EA), Other allele (OA).

File Name: Supplementary Data 9

Description: Functional annotation of all variants down to r^2 > 0.4 of MM sentinel SNPs along with their annotation from Micro-C, ChromHMM, GWAS data, new MPRA, published MPRA, and luciferase assays. GM12878 and Bone Marrow mesenchymal stem cell (BMSC) chromHMM tracks are from Roadmap Epigenomics data. Potential enhancer: correlated variant resides in enhancer in any cell type; Potential enhancer with looping: potential enhancer which also shows looping to a gene in any cell type; Potential TSS: correlated variant resides in ChromHMM defined TSS and is proximal to TSS of a gene. Reporter assays are shown with respect to the alternate allele i.e. if log2 fold-change or alt-ref is positive, the alternate allele increases transcription. TF, transcription factor; logFC, log2 Fold Change; RA, risk allele; OR, odds ratio.

File Name: Supplementary Data 10

Description: Expression quantitative trait loci in high LD with MM risk variants (r^2 > 0.8 between MM lead variant and eQTL lead variant). When nominating target genes based on effects on expression, we primarily considered eQTLs in the B-cell lineage (i.e., in plasma cells, plasmablasts, and B-cell populations). If no effect on expression was detected in the B-cell lineage, we accepted eQTLs in other hematologic lineages.

File Name: Supplementary Data 11

Description: Target genes showing essentiality in hematologic cell lines in two DepMap data sets: CRISPR/Cas9 DepMap Public 23Q2 (pooled CRISPR/Cas9 proliferation screens for 1,092 cell lines) and RNAi Achilles+DRIVE+Marcotte (pooled RNAi proliferation screens for 710 cell lines). P-values for enrichment for negative perturbation scores: Chronos scores for CRISPR data; DEMETER2 scores for RNAi data. Data from www.depmap.org. Plasma cell myeloma lines highlighed in dark green; other lymphoid cell lines in light green.

File Name: Supplementary Data 12

Description: Gene Ontology (GO) and UniProt categories with significant enrichment of MM target genes (STRING database; FDR < 5%; background gene count > 10).

File Name: Supplementary Data 13

Description: Colocalisation analysis for LTL with MM at genome-wide significant MM loci. The coloc package evaluates four possible configurations of causal variants for two traits. H0: No association with either trait, H1: Association with trait 1, but not trait 2, H2: Association with trait 2, but not trait 1, H3: Association with trait 1 and 2, but independent variants, H4: Association with trait 1 and 2, shared variant (indicated in green). Posterior probability > 0.80 was considered as supporting a specific model.

File Name: Supplementary Data 14

Description: Summary statistics for LTL variants used for Mendelian randmization analysis. The variants listed showed significant association with LTL in the UK Biobank data set, and could also be successfully mapped to the MM meta-analysis. Abbreviations: EA, effect allele; EAF, effect allele frequency; OA, other allele; SE, standard error.

File Name: Supplementary Data 15

Description: Causal estimates from the Mendelian randomisation analysis for each exposure and MM risk. BCMA_SomaScan and IL5RA_SomaScan represent the replication analysis in the Icelandic SomaScan dataset. Abbreviations: BCMA, B-cell maturation antigen; LTL, leukocyte telomere length; MR, Mendelian randomisation; SE, standard error.

File Name: Supplementary Data 16

Description: Results of Mendelian randomization sensitivity analyses. Pleiotropy (MR Egger regression), heterogeneity and Steiger test sensitivity analysis for exposure versus MM risk. Abbreviations: IVW, inverse variance-weighted; MR, Mendelian randomization.

File Name: Supplementary Data 17

Description: Association statistics for UK Biobank Olink *trans*-pQTLs in high LD ($r^2 > 0.8$ between pQTL lead variant and MM lead variant). Also shown are summary statistics for the replication analysis in the Icelandic SomaScan proteomics data set.

File Name: Supplementary Data 18

Description: Summary statistics for *trans*-pQTL variants used in MR analysis. The listed variants are genome-wide significant lead variants for serum levels of their respective

proteins in the UK Biobank OLink data. BCMA_SomaScan and IL5RA_SomaScan represents data used for the replication in SomaScan dataset. Abbreviation: EA, effect allele; EAF, effect allele frequency; OA, other allele; SE, standard error.

File Name: Supplementary Data 19

Description: Results for colocalization analysis for pQTLs with MM at genome-wide significant MM loci. The coloc package evaluates four possible configurations for two traits. H0: No association with either trait, H1: Association with trait 1, but not trait 2, H2: Association with trait 2, but not trait 1, H3: Association with trait 1 and 2, but independent variants, H4: Association with trait 1 and 2, shared variant (indicated in green). Posterior probability > 0.80 was considered as supporting a specific model.

File Name: Supplementary Data 20

Description: Wald ratio estmates for each variant. Abbreviations: SE, standard error.

File Name: Supplementary Data 21

Description: Results of MR analysis excluding one variant at a time (leave-one-out analysis, LOO). This tests whether a single variant is driving a positive association. Abbreviations: SE, standard error.

File Name: Supplementary Data 22

Description: Protein quantitative trait loci (pQTLs) for variants-of-interest at TNFRSF13B in the UK Biobank data. Abbreviations: CVID, common variable immunodeficiency; EA, effect allele; EAF, effect allele frequency; MM, multiple myeloma; OA, other allele.

File Name: Supplementary Data 23

Description: Primers used in the massively-parallel reporter assay (MPRA) experiments.

File Name: Supplementary Data 24

Description: sgRNA sequences and primers used in the dual-sgRNA CRISPR/Cas9 deletion experiments.

File Name: Supplementary Data 25

Description: Quantitative PCR assays used to quantify *TNFRSF13B* expression in the dual-sgRNA CRISPR/Cas9 deletion experiments.
