# Supplementary material for: Deciphering the genetics and mechanisms of predisposition to multiple myeloma
Source: Nat Commun. 2024 Aug 5;15:6644. doi: 10.1038/s41467-024-50932-7 (PMC11300596; doi:10.1038/s41467-024-50932-7)
Supplement: Supplementary file 5 — Reporting Summary [file 41467_2024_50932_MOESM5_ESM.pdf]

nature portfolio

Corresponding author(s): NCOMMS-23-58723-T

Last updated by author(s): 2024, 01-09

Reporting Summary

Nature Portfolio wishes to improve the reproducibility of the work that we publish. This form provides structure for consistency and transparency in reporting. For further information on Nature Portfolio policies, see our Editorial Policies and the Editorial Policy Checklist.

Statistics

For all statistical analyses, confirm that the following items are present in the figure legend, table legend, main text, or Methods section.

n/a	Confirmed	
☐	☒	The exact sample size (n) for each experimental group/condition, given as a discrete number and unit of measurement
☐	☒	A statement on whether measurements were taken from distinct samples or whether the same sample was measured repeatedly
☐	☒	The statistical test(s) used AND whether they are one- or two-sided<br>*Only common tests should be described solely by name; describe more complex techniques in the Methods section.*
☐	☒	A description of all covariates tested
☐	☒	A description of any assumptions or corrections, such as tests of normality and adjustment for multiple comparisons
☐	☒	A full description of the statistical parameters including central tendency (e.g. means) or other basic estimates (e.g. regression coefficient) AND variation (e.g. standard deviation) or associated estimates of uncertainty (e.g. confidence intervals)
☐	☒	For null hypothesis testing, the test statistic (e.g. F, t, r) with confidence intervals, effect sizes, degrees of freedom and P value noted<br>*Give P values as exact values whenever suitable.*
☒	☐	For Bayesian analysis, information on the choice of priors and Markov chain Monte Carlo settings
☒	☐	For hierarchical and complex designs, identification of the appropriate level for tests and full reporting of outcomes
☐	☒	Estimates of effect sizes (e.g. Cohen's d, Pearson's r), indicating how they were calculated

Our web collection on statistics for biologists contains articles on many of the points above.

Software and code

Policy information about availability of computer code

Data collection: No software was used for data collection

Data analysis: We used publicly available software (URLs listed below) in conjunction with the above described algorithms in the sequencing processing pipeline (Whole-genome sequencing, Association testing, RNA-seq mapping and analysis):

Graphtyper https://github.com/DecodeGenetics/graphtyper
Eagle https://alkesgroup.broadinstitute.org/Eagle/
ADMIXTURE v1.23 http://www.genetics.ucla.edu/software
PLINK v.190b3a http://pngu.mgh.harvard.edu/purcell/plink/
UMAP https://github.com/diazale/umap_review
GORpipe https://github.com/gorpipe/gor
UCSC Browser https://genome.ucsc.edu/
COLOC software package https://cran.r-project.org/web/packages/coloc/vignettes/a01_intro.html
GWAS catalog https://www.ebi.ac.uk/gwas/
Further, R version 3.6.0 was used to analyze data and create plots.

For manuscripts utilizing custom algorithms or software that are central to the research but not yet described in published literature, software must be made available to editors and reviewers. We strongly encourage code deposition in a community repository (e.g. GitHub). See the Nature Portfolio guidelines for submitting code & software for further information.

Data

Policy information about availability of data

All manuscripts must include a data availability statement. This statement should provide the following information, where applicable:

- Accession codes, unique identifiers, or web links for publicly available datasets
- A description of any restrictions on data availability
- For clinical datasets or third party data, please ensure that the statement adheres to our policy

Sequence variants passing GATK filters have been deposited in the European Variation Achive, accession number PRJEB15197 (https://www.ebi.ac.uk/ena/data/view/PRJEB15197). The authors declare that the data supporting the findings of this study are available within the article, it supplementary files, and upon request.

Research involving human participants, their data, or biological material

Policy information about studies with human participants or human data. See also policy information about sex, gender (identity/presentation), and sexual orientation and race, ethnicity and racism.

Reporting on sex and gender	In the association analysis, we adjusted for sex determined from genotype data.
Reporting on race, ethnicity, or other socially relevant groupings	In the association analysis, we used data on individuals of European descent. We used linkage disequilibrium (LD) score regression to account for distribution inflation in the dataset due to cryptic relatedness and population stratification.
Population characteristics	The Icelandic dataset is based on whole-genome sequence data from 63,460 Icelanders participating in various research projects at deCODE genetics. Variants identified through whole-genome sequencing were imputed into 173,025 chip-genotyped Icelanders as well as their untyped close relatives based on genealogy. The Swedish dataset is based on whole-genome sequence data of 17,408 individuals of European origin, including 3,704 Swedes participating in various research projects at deCODE. The sequence genotypes were phased with chip genotypes and 170 million variants identified in the whole-genome sequencing were imputed into the phased chip data.
Recruitment	For the Icelandic and Swedish dataset individuals were recruited through various research projects at deCODE genetics. Specific for this project, the IgG subclass concentrations for the Icelanders were obtained from clinical laboratories for 2,585 adults aged 18 to 92 years, as well as for 4,371 children and adolescents under 18 years. To generate the Swedish data set, we measured IgG subclass concentrations in 1,749 adult blood donors.
Ethics oversight	The study was approved by the National Bioethics Committee in Iceland (Approval no. 15-023) following a review by the Icelandic Data Protection Authority. All participating subjects who donated blood signed informed consent. The personal identities of the participants and biological samples were encrypted using a third-party system approved and monitored by the Icelandic Data Protection Authority. The Swedish samples were collected subject to ethical approval (Lund University Ethical Review Board, dnr 2013/54).

Note that full information on the approval of the study protocol must also be provided in the manuscript.

Field-specific reporting

Please select the one below that is the best fit for your research. If you are not sure, read the appropriate sections before making your selection.

☒ Life sciences ☐ Behavioural & social sciences ☐ Ecological, evolutionary & environmental sciences

For a reference copy of the document with all sections, see nature.com/documents/nr-reporting-summary-flat.pdf

Life sciences study design

All studies must disclose on these points even when the disclosure is negative.

Sample size	The samples size corresponds to the available data from Iceland and Sweden
Data exclusions	Icelandic individuals with known diagnosis of multiple myeloma, nonoclonal gammopathy of unknown significance, Waldenström's disease, lymphoid malignancies, liver cirrhosis or primary biliary cirrhosis in deCODE's phenotype database were excluded. No other available data was excluded from the analyzes.
Replication	We performed GWAS studies in two independent populations and combined the results. Results are presented for the populations independently and combined and heterogeneity of effects between gopus are assessed.
Randomization	Not relevant for this study
Blinding	Not relevant for this study

Reporting for specific materials, systems and methods

We require information from authors about some types of materials, experimental systems and methods used in many studies. Here, indicate whether each material, system or method listed is relevant to your study. If you are not sure if a list item applies to your research, read the appropriate section before selecting a response.

Materials & experimental systems

n/a	Involved in the study
☐	☒ Antibodies
☐	☒ Eukaryotic cell lines
☒	☐ Palaeontology and archaeology
☒	☐ Animals and other organisms
☒	☐ Clinical data
☒	☐ Dual use research of concern
☒	☐ Plants

Methods

n/a	Involved in the study
☒	☐ ChIP-seq
☐	☒ Flow cytometry
☒	☐ MRI-based neuroimaging

Antibodies

Antibodies used	EasySep magnetic cell sorting (StemCell Technolocies, cat. # 19674, 19662, and 19663 respectively)
Validation	EasySep magnetic cell sorting is quality control tested by the manufacturer as stated on their website https://www.stemcell.com/

Eukaryotic cell lines

Policy information about cell lines and Sex and Gender in Research

Cell line source(s)	different plasma cell lines (MOLP8, L363 and RPMI8226), HEK cells
Authentication	*Describe the authentication procedures for each cell line used OR declare that none of the cell lines used were authenticated.*
Mycoplasma contamination	*Confirm that all cell lines tested negative for mycoplasma contamination OR describe the results of the testing for mycoplasma contamination OR declare that the cell lines were not tested for mycoplasma contamination.*
Commonly misidentified lines (See ICLAC register)	*Name any commonly misidentified cell lines used in the study and provide a rationale for their use.*

Plants

Seed stocks	NA
Novel plant genotypes	NA
Authentication	NA

Flow Cytometry

Plots

Confirm that:

☒ The axis labels state the marker and fluorochrome used (e.g. CD4-FITC).

☒ The axis scales are clearly visible. Include numbers along axes only for bottom left plot of group (a 'group' is an analysis of identical markers).

☒ All plots are contour plots with outliers or pseudocolor plots.

☒ A numerical value for number of cells or percentage (with statistics) is provided.

Methodology

Sample preparation	*Describe the sample preparation, detailing the biological source of the cells and any tissue processing steps used.*
Instrument	*Identify the instrument used for data collection, specifying make and model number.*
Software	*Describe the software used to collect and analyze the flow cytometry data. For custom code that has been deposited into a community repository, provide accession details.*
Cell population abundance	*Describe the abundance of the relevant cell populations within post-sort fractions, providing details on the purity of the samples and how it was determined.*
Gating strategy	*Describe the gating strategy used for all relevant experiments, specifying the preliminary FSC/SSC gates of the starting cell population, indicating where boundaries between "positive" and "negative" staining cell populations are defined.*

☐ Tick this box to confirm that a figure exemplifying the gating strategy is provided in the Supplementary Information.
